# Innate Sensing of the Gut Microbiota: Modulation of Inflammatory and Autoimmune Diseases

**DOI:** 10.3389/fimmu.2016.00054

**Published:** 2016-02-19

**Authors:** Aline Ignacio, Camila Ideli Morales, Niels Olsen Saraiva Câmara, Rafael Ribeiro Almeida

**Affiliations:** ^1^Laboratory of Transplantation Immunobiology, Department of Immunology, Institute of Biomedical Sciences, University of São Paulo, São Paulo, Brazil; ^2^Department of Medicine, Nephrology Division, Federal University of São Paulo, São Paulo, Brazil; ^3^Renal Pathophysiology Laboratory, Department of Clinical Medicine, University of São Paulo, São Paulo, Brazil

**Keywords:** microbiota, toll-like receptors, inflammasome, autoimmunity, inflammatory diseases

## Abstract

The mammalian gastrointestinal tract harbors a diverse microbial community with which dynamic interactions have been established over millennia of coevolution. Commensal bacteria and their products are sensed by innate receptors expressed in gut epithelia and in gut-associated immune cells, thereby promoting the proper development of mucosal immune system and host homeostasis. Many studies have demonstrated that host–microbiota interactions play a key role during local and systemic immunity. Therefore, this review will focus on how innate sensing of the gut microbiota and their metabolites through inflammasome and toll-like receptors impact the modulation of a distinct set of inflammatory and autoimmune diseases. We believe that a better understanding of the fine-tuning that governs host–microbiota interactions will further improve common prophylactic and therapeutic applications.

## Introduction

The mammalian gastrointestinal (GI) tract harbors more than 500 bacterial species that have protective, metabolic, and trophic roles and are a constant source of stimulation for the immune system (1). Surveillance mechanisms of the innate immune system control the communication of the gut microbiota with the internal environment, preventing their penetration and systemic spread, and maintaining intestinal homeostasis.

The GI innate immune system consists primarily of a physical barrier, which is composed of intestinal epithelial cells (IECs), represented by absorptive enterocytes, mucus-producing goblet cells, hormone-producing enteroendocrine cells, and Paneth cells, which produce antimicrobial peptides and lectins, among other molecules. The selective permeability of the epithelial barrier allows nutrients absorption while contributing to the immune responses by providing microbial products, which can be recognized through pattern recognition receptors (PRRs), including toll-like receptors (TLRs), nucleotide-binding domain leucine-rich repeat-containing receptors (NLRs), RIG-I like receptors (RLRs), C-type lectin family, and AIM2-like receptors (ALRs) (2), triggering different intracellular signaling cascades against pathogen-associated molecular patterns (PAMPs) or damage-associated molecular patterns (DAMPs).

The relationship between the intestinal epithelium and the gut microbiota is not restricted to prevent host invasion by commensals. Bacteria-mediated fermentation of food that cannot be fully digested in the mammalian intestine releases products such as short-chain fatty acids (SCFAs), which are used as fuel by IECs. In turn, the host provides a stable, nutrient-rich ecosystem for commensals to thrive. The microfold (M) cells are specialized epithelial cells of the mucosa-associated lymphoid tissue found in the follicle-associated epithelium (FAE) that mediate the uptake and transepithelial transport of luminal antigens to intraepithelial immune cells, such as macrophages and dendritic cells, which are responsible for presenting antigens to lymphocytes (3). In the intestinal lamina propria, macrophages and dendritic cell-mediated antigen uptake, either directly from the intestinal lumen by extending projections between IECs or indirectly by M cells, results in cytokine production, which drives either inflammatory Th17 or regulatory T cells, and T-cell-dependent and -independent IgA class-switching responses (4–6).

In this review, we focused on the interactions between the gut microbiota and two distinct groups of molecules, the innate sensors, namely, TLRs and the inflammasomes, which are typically composed of the innate sensors NLRs and ALRs, the adaptor protein apoptosis-associated speck-like protein containing a caspase-recruitment domain (ASC), and the proinflammatory caspase-1. Increasing efforts have been pointed out toward the understanding of how innate sensing of commensals promotes homeostasis and immunity. Here, we review the impact of innate sensing of the gut microbiota on inflammatory and autoimmune diseases’ outcomes.

## Toll-Like Receptors

Toll-like receptors are type I transmembrane proteins with an extracellular horseshoe-shaped domain-containing leucine-rich repeats (LRRs) and a cytoplasmic tail containing a toll/IL-1 receptor (TIR) domain (7). They correspond to one of the five main families of PRRs, which recognize a variety of PAMPs and DAMPs.

Toll-like receptors are capable of forming both homodimers and heterodimers to detect different types of ligands. TLR2/TLR1 heterodimers recognize triacylated lipopeptides (8), and it has been recently demonstrated that they can also be activated by α-synuclein, a protein associated with Parkinson’s disease (9). On the other hand, diacylated lipopeptides are recognized by TLR2/TLR6 heterodimers (8).

A recent study has shown that human immunodeficiency virus (HIV-1) structural proteins can activate TLR2- and TLR2-related heterodimers (10). A variety of viral proteins from measles virus (MV), hepatitis C virus (HCV), human cytomegalovirus (hCMV), and herpes simplex (HSV) have also been indicated as TLR2 activators (11–14). Lipoteichoic acid (LTA), zymosan, and peptidoglycan also trigger TLR2 signaling when TLR2/TLR6/CD14, TLR2/Dectin-1, and TLR2/CD14 are assembled, respectively (15–20).

TLR3 recognizes viral double-stranded RNA (dsRNA) (21) and is also capable of recognizing DAMPs released from damaged tissues (22), while TLR7 and TLR8 detect single-stranded RNA (ssRNA) found during viral replication (23–25). TLR4 is the main sensor for lipopolysaccharides (LPS) from Gram-negative bacteria (26) and also detects viral motifs (27). Flagellin is sensed by TLR5 (28), and TLR9 detects unmethylated CpG sequences in DNA molecules of bacterial and viral genomes (29). TLR10 is expressed in humans, but not in mice, making the search for a specific ligand experimentally difficult. It has been recently shown that TLR10 expressed in IECs responds to an unidentified component of *Listeria monocytogenes*, but it requires the presence of TLR2 for inducing NFκB activation (30). This opens the possibility that TLR10 acts as a coreceptor of TLR2, as it is phylogenetically related to TLR1 and TLR6 (31). Furthermore, other authors have demonstrated anti-inflammatory properties of TLR10 expressed in human peripheral blood mononuclear cells (PBMCs) (32).

TLR11 is expressed in mice and as a non-functional pseudogene in humans and has been related to prevention of *Salmonella* infection, the detection of uropathogenic *Escherichia coli*, and the recognition of a profiling-like molecule from *Toxoplasma gondii* (33–35). TLR12 and TLR13 are not expressed in humans but are expressed in mice and are capable of detecting the profiling-like molecule of *T. gondii* and 23S rRNA, respectively (36, 37). Besides PAMPs, endogenous ligands activate TLRs in the absence of infection (38).

Toll-like receptor–ligand interactions lead to dimerization of extracellular domains and results in dimerization of their cytoplasmic TIR domains. This structural modification is recognized by TIR domains on the adaptor proteins myeloid differentiation primary response gene 88 (MyD88), MyD88 adapter-like (MAL)/TIR domain-containing adapter protein (TIRAP), TIR domain-containing adapter-inducing interferon-β (TRIF), and TRIF-related adaptor molecule (TRAM), triggering downstream signaling pathways, which culminate in the expression of inflammatory cytokines, several anti-viral and anti-pathogen proteins, leading to the initiation of the adaptive immune response. The Myd88-dependent signaling pathway is common to all TLRs, except TLR3, which is dependent on TRIF. TLR4 uses both MyD88- and TRIF-dependent signaling pathways (7).

The detection of common molecules shared by pathogenic and non-pathogenic bacteria should trigger the same inflammatory response, but paradoxically, the recognition of commensal bacteria by intestinal receptors is somehow regulated to not result in inflammatory responses but to induce tolerance. Moreover, these TLR-mediated signals contribute to the intestinal homeostasis by regulating IECs proliferation and epithelial integrity (39–42). Interestingly, the localization, distribution, and expression of TLRs in the intestinal epithelium seem to be directly related to their role in maintaining homeostasis (43). IECs have a polarized organization, with an apical pole facing the intestinal lumen and a basolateral one that communicates with other IECs and the lamina propria. TLRs are differentially distributed through the polarized organization of IECs and in different quantities along the entire GI tract.

The human esophagus expresses TLR4, low levels of TLR1 and TLR5, and high levels of TLR2 and TLR3 mRNA in epithelial cells (44, 45). In gastric epithelial cells, it is possible to find expression of TLR2, TLR4, and TLR5, as both mRNA and protein (46). In the human small intestine, it is possible to find mRNA expression from the TLRs 1–10 (47). Under homeostatic conditions, enteroendocrine IECs from intestinal crypts express TLR1, TLR2, and TLR4 (48), and IECs from human colon express relatively high levels of mRNA for TLR3 and less for TLR2 and TLR4 (49).

Colonic IECs are hyporesponsive to LPS, and it may be due to epigenetic mechanisms that downregulate MD-2 and TLR4 expression (50). Moreover, TLR3 in murine IECs seems to have an age-dependent expression related to an enhanced response to rotavirus in adult mice (51). On the other hand, the high basolateral expression of TLR5 is directly induced by administration of flagellin to IECs (52). TLR9 apical expression in colonic IECs has been related to the maintenance of the intestinal ­homeostasis (53).

The FAE presents a differential expression of TLRs in comparison with villous IECs. The murine small intestine expresses TLR2 and TLR9 in both apical and basolateral sides of the FAE, whereas IECs in the villi shows apical expression only. The TLR5 is abundantly expressed in the apical pole of villous IECs and FAE, whereas TLR4 expression is low (54).

It is conceived that TLR-mediated sensing of microbial products has a dual role in promoting the fine-tune balance between proinflammatory and pro-regulatory immune responses. A better understanding of how these molecules are regulated in face of commensals and pathogenic microorganisms will benefit the design of prophylactic and therapeutic approaches.

## The Dual Role of TLRs in Triggering Inflammatory and Autoimmune Diseases

Bearing in mind the importance of the gut microbiota for the development of the immune system, one might speculate that microbial components could also modulate immunity and trigger inflammatory and autoimmune diseases. Several studies have shown correlations between altered expression of TLRs and inflammatory/autoimmune conditions, and increasing evidence suggests the participation of the gut microbiota during TLRs abnormal signaling and the development of diseases, including type 1 diabetes (T1D), multiple sclerosis (MS), rheumatoid arthritis (RA), and inflammatory bowel diseases (IBDs) (Table 1) (55–58).

**Table 1:** **Toll-like receptors and related molecules involved in inflammatory and autoimmune diseases**.

Molecule	Disease	Model	Evidence	Reference
IL-1 receptor, TLR2, TLR4	Rheumatoid arthritis (RA)	*IL1rn*^−/−^	TLR2 deficiency in *IL1rn*^−/−^ mice led to less Foxp3 expression and reduced suppressive activity of Tregs; *IL1rn*^−/−^*Tlr4*^−/−^ mice were protected	(56)
		Germ-free mice		
TLR2		DBA-1 mice	Repeated oral inoculations of the periodontal pathogens *Porphyromonas gingivalis* and *Prevotella nigrescens*-induced Th17-mediated periodontitis in mice, which was dependent on TLR2-expressing APCs	(74)
TLR2, MyD88	Inflammatory bowel disease (IBD)	C57Bl/6 mice	Variants of the MDR1/ABCB1 gene have been associated with susceptibility to UC. TLR2/MDR1A double-knockout mice presented exacerbated colitis score, which could be inhibited by treatment with IL-1R antagonist; intestinal CD11b^+^ Ly6C^+^-derived IL-1β production and inflammation was dependent on MyD88	(77)
TLR2, TLR4, MyD88		129/SvJ × C57Bl/6 mice	TLR2^−/−^, TLR4^−/−^, and MyD88^−/−^ mice showed increased susceptibility to colonic injury than WT. Antibiotic treatment increased mortality and morbidity, and abrogated the production of cytoprotective and reparative factors	(39)
TLR2, TLR3, TLR4, TLR5		Human; adults	TLR2 and TLR5 expression on IECs remain unchanged in active IBD; upregulation of TLR2 was observed in inflammatory cells from the lamina propria; UC and CD patients showed differential expression of TLR3 and TLR4, which occurred on basolateral and apical surfaces of IEC	(81)
TLR4		Human; adults	DCs from UC and CD patients showed increased TLR4 expression and the uptake of LPS started earlier than in controls; stimulated DCs secreted high amounts of TNF-α and IL-8	(83)
TLR9		C57Bl/6 mice	Apical TLR9 stimulation on IECs conferred intracellular tolerance to subsequent TLR challenges; IECs from TLR9-deficient mice displayed lower NF-κB activation threshold, and these mice were highly susceptible to experimental colitis	(53)
TLR9, TLR3	Type 1 diabetes (T1D)	BioBreeding Diabetes Resistant (BBDR) rats	ssDNA parvovirus Kilham rat virus (KRV) acts as a TLR9 ligand to upregulate proinflammatory cytokines and induce islet destruction; pretreatment with poly I:C acts synergistically with KRV to induce diabetes in 100% of infected rats	(90)
TLR2, TLR4		NOD mice	Apoptotic β-cell is sensed by APCs through TLR2, which could stimulate the priming of diabetogenic T cells	(91)
MyD88		NOD mice	MyD88-deficient NOD mice did not develop T1D	(58)
		Germ-free mice	Germ-free (GF) MyD88-deficient NOD mice developed T1D; colonization of GF MyD88-deficient NOD mice with the bacterial community present in healthy mouse gut-attenuated symptoms	
MyD88, TRIF, TLR2, TLR4		NOD mice	TRIF deficiency did not promote T1D development in MyD88 sufficient NOD mice; only double-deficient mice were susceptible to T1D; reduction in disease incidence caused by TLR2 deletion was reversed in GF TLR2-deficient mice	(92)
		Germ-free mice		
TLR4, TLR9, MyD88	Multiple sclerosis (MS)	C57Bl/6 mice	LPS- and CpG-stimulated B cells produce IL-10 in a MyD88-dependent manner; DCs produce less IL-12 and restrain Th1 differentiation	(102)
TLR4		C57Bl/6 mice	TLR4 is highly expressed in Th17 cells and LPS directly stimulated Th17 differentiation *in vitro*; subcutaneous injection of LPS increased the frequency of IL-17 producing cells worsening experimental autoimmune encephalomyelitis (EAE)	(100)

*IL1rn, IL-1 receptor antagonist-deficient mice; MDR1, multidrug resistance gene; UC, ulcerative colitis; IECs, intestinal epithelial cells; CD, Crohn’s disease*.

Rheumatoid arthritis, IBDs, T1D, and MS are classified as multifactorial diseases with genetic and environmental factors contributing to their pathogenesis. Among the environmental factors, the exposition to microbial components has been associated with their development (55, 59–62). The concept called “molecular mimicry” was proposed to explain the role of microbial agents in inflammatory and autoimmune diseases, and according to this concept, cross-reactivity between epitopes from microbes and self-proteins can cause deregulated immune responses, leading to autoantibody production and activation of effectors cells (63).

The major organs exposed to microbial agents and their components are the skin and gut. In the gut, sensing of microbial antigens by innate immune cells and non-immune cells, such as IECs and stromal cells, is mediated by PRRs that recognized PAMPs. It is possible that bacterial components stimulate the mucosal immune cells by penetrating a damaged epithelial barrier or *via* a paracellular pathway; alternatively, microbial antigens may interact with TLRs at the apical surface of IECs, inducing inflammatory activation of the mucosal immune system. In addition, innate immune cells, such as dendritic cells and macrophages, can also sense PAMPs through TLRs initiating rapid and effective inflammatory responses against microbial invasion (57, 64). As mentioned above, the commensal microbiota is necessary for the constant stimulation of the immune system (1) and TLR-mediated sensing of these microorganisms may play a dual role in disease development as a source of both inflammatory and regulatory signals.

Rheumatoid arthritis is an autoimmune disease characterized by chronic inflammation in which cartilage and bone of the affected subjects are progressively destroyed in multiple joints (55). Several immune and resident cells, such as chondrocytes and fibroblasts, contribute to the development and progression of RA. Release of proinflammatory cytokines, such as tumor-necrosis factor (TNF)-α and interleukin-1 (IL-1), mainly by macrophages, activation of Th17 lymphocytes, and production of autoantibodies are suggested to play an important role in the disease onset (65). Studies showing the presence of bacterial cell wall components in the joints of RA patients accompanied by changes in their gut microbiota support the idea that commensal bacteria may initiate inflammation in genetically susceptible individuals (66, 67).

Abdollahi-Roodsaz et al. (56) using the IL-1 receptor antagonist-deficient (*IL1rn*^−/−^) mice that spontaneously develop T-cell-mediated autoimmune arthritis showed that germ-free (GF) *IL1rn*^−/−^ presented no signs of arthritis during 20 weeks of follow-up, whereas matched non-GF animals started to develop the disease from the age of 5 weeks. The authors also demonstrated that the activation of TLRs was dependent on the microbial status of the mice and that TLR2 deficiency in *IL1rn*^−/−^ mice led to less Foxp3^+^ expression and reduced suppressive activity of Tregs, resulting in an enhancement of clinical and histopathological scores of arthritis. By contrast, *IL1rn*^−/−^*Tlr4*^−/−^ mice were protected against severe disease. It has been shown that TLR4 contributes to more severe arthritis by modulating IL-17 production and Th17 cell expansion (56, 68). In fact, gut-residing segmented filamentous bacteria (SFB) have been shown to drive both IL-1β and IL-6 production, and Th17 development, which promotes arthritis (69, 70). Therefore, one may conceive that a delicate equilibrium in TLR-mediated sensing of the gut microbiota is necessary to maintain homeostasis and prevent certain autoimmune diseases.

Although the gut microbiota has been extensively explored as an etiologic factor of RA, less is known about oral commensal microorganisms. In this context, the Gram-negative commensal bacterium *Porphyromonas gingivalis* has been a major focus of investigation as it provides a direct link between a specific microorganism and an autoimmune disease (71, 72). In fact, individuals with periodontitis present an increased proportion of *P. gingivalis* and specific antibodies to this bacterium, which has been linked to RA (73). A recent study showed that periodontal disease (PD) induced by *P. gingivalis* increases the severity of experimental arthritis through Th17 induction. This periodontal pathogen induces IL-17-producing T cells through TLR2 activation on APCs (74), which suggests that the same TLR molecule may play different roles in RA onset, depending on where the molecule is expressed and with which microorganism the interaction occurs. Thus, more studies are still necessary to better determine the role of TLR-mediated sensing of the microbiota and/or pathogens in autoimmune diseases, such as RA. Mice models with organ-specific TLR expression could be used to shed light on how TLRs contribute to disease onset depending on their localization.

Inflammatory bowel diseases have been extensively studied in regarding of the gut microbiota–immune system interactions. Two major forms of IBD have been investigated: the ulcerative colitis (UC) and Crohn’s disease (CD). Distinctive and complex chronic inflammatory processes characterize both disorders (61, 62), and it has been demonstrated that the intestinal microbiota of patients differ from healthy controls, showing an increase of *Enterococcus* spp. and *Bacteroides* spp. accompanied by a decrease of *Bifidobacterium* spp. and *Lactobacillus* spp. levels (75, 76), suggesting that the gut microbiota may play a pivotal role in intestinal inflammatory diseases.

Increased susceptibility to severe UC has been associated with variants of the multidrug resistance gene (MDR1/ABCB1). Deletion of TLR2 in MDR1A deficiency resulted in fulminant pancolitis, characterized by expansion of CD11b^+^ myeloid cells, and a shift toward Th1 immune responses in the lamina propria. An unaltered microbiota was required for colitis exacerbation in TLR2/MDR1A double-knockout mice once protection from colitis was observed upon antibiotic treatment (77). Treg-specific deletion of MyD88 has been recently shown to culminate in deficiency of Treg cells, increase in Th17 cells, and exacerbated experimental colitis. An overgrowth of SFB and increased bacterial translocation was reported, and also impaired antimicrobial IgA responses (78), suggesting that the TLR-mediated sensing of the gut microbiota contributes to Treg cell-dependent maintenance of the intestinal homeostasis. On the other hand, it has been shown that gut microbiota-mediated triggering of intestinal epithelial TLRs is not critical for promoting intestinal inflammation once mice lacking TNF receptor-associated factor 6 (TRAF6), but not MyD88/TRIF, were protected from dextran sodium sulfate (DSS)-induced colitis (79). Therefore, it is becoming clear that not only the microbiota is important during TLRs signaling through colitis development but also where these molecules are expressed.

Apart from the alterations in the gut microbiota composition, Cario and Podolsky have studied TLRs expression in CD and UC patients and showed variations in the expression of some receptors. During homeostasis, TLR2 and TLR4 are presented in small amounts on IECs and lamina propria cells to minimize microbiota recognition and to maintain tolerance (80, 81). So, signaling through those PRRs may determine the balance between immunity and tolerance. The authors observed that TLR2 and TLR5 are expressed on IECs from non-IBD subjects and remain unchanged in active IBD. Upregulation of TLR2 was observed in inflammatory cells from the lamina propria of active IBD patients; differential expression of TLR3 was verified in UC patients, which presented a basolateral expression on IECs; both UC and CD patients showed an abundant TLR4 expression on basolateral and apical surfaces of IECs, respectively, and enhanced expression of TLR4 was also present in the lamina propria of IBD individuals (80).

In DCs, PRRs stimulation induces IL-23 releases, which is an important component of antimicrobial defense, but when excessively produced it favors proinflammatory T-cell response and reduces Foxp3^+^ T cell differentiation. In this way, DCs may act as a key player in the initiation, continuation, and control of IBD (82). Conventional DCs (cDC) from UC and CD patients during remission phase showed an increased TLR4 expression. After stimulation, these cells secreted higher amounts of TNF-α and IL-8, and the uptake of LPS started earlier and was higher than in controls (83). These data suggest that an aberrant TLR4 signaling in cDC of IBD patients may result in an inflammatory phenotype during the acute phase.

Paradoxical effects of TLRs activation may be present in IBD. Bacterial DNA induces strong Th1 immune responses with high production of TNF-α and IL-8, which is also found in experimental and human IBD (84). On the other hand, bacterial DNA stimulation of IECs *via* apical TLR9 may result in anti-inflammatory effects, with the inhibition of TNF-α and IL-8 secretion, as well as NFκB activation, reducing colitis severity (53). Furthermore, it has been observed in a CD4^+^ T-cell-dependent Severe combined immunodeficiency (SCID) transfer model of colitis that pretreatment of donor mice with CpG completely abolished colitis development in SCID recipients in a CpG–TLR9-mediated modulation of T-cell function (85).

Another important protective effect promoted by TLR signaling was recently elucidated by Kawashima et al. (86) showing that dsRNA of lactic acid bacteria (LAB), one major commensal bacteria, triggered interferon-β (IFN-β) production through TLR3 activation pathway and protected mice from experimental colitis. On the other hand, pathogenic bacteria induced much less IFN-β and contained less dsRNA than LAB, indicating that dsRNA was not involved in pathogen-induced IFN-β induction. These results point toward TLR3 as a sensor to commensal bacteria and suggest a mechanism by which this endosomal receptor contributes to anti-inflammatory and protective immune responses.

Paradoxical effects of TLRs participation are also observed in T1D, an organ-specific autoimmune disease in which insulin-producing β cells are mainly destroyed by not only Th1 but also Th17 lymphocytes (87). The autoreactive CD4^+^ T cells that infiltrate the pancreas present a proinflammatory phenotype, characterized by IFN-γ secretion (88), and support cytotoxic T lymphocytes (Tc), which are responsible for the progressive destruction of β cells (55). Although the later steps of T1D development are well known, the initial steps remain unclear.

The role of TLRs–PAMP interactions during T1D onset has been investigated in several animal models. Studies performed in BioBreeding Diabetes Resistant (BBDR) rats infected with the ssDNA parvoviruses Kilham rat virus (KRV) showed that KRV acts as a TLR9 ligand by upregulating proinflammatory cytokines in pancreatic lymph nodes, thus inducing islet destruction (89). Pretreatment with poly I:C, a TLR3 ligand, acts synergistically with KRV to induce diabetes in 100% of infected rats (90). Also, it has been demonstrated that the activation of APCs through TLR2-mediated sensing of β cells death contributes to T1D initiation in non-obese (NOD) mice (91). In contrast with these findings, Wen et al. (58) investigating the effects of *MyD88* gene disruption on disease incidence and progression in NOD mice showed that MyD88-deficient NOD mice did not develop T1D, and that the observed protection was dependent on commensal microorganisms once GF MyD88-deficient NOD mice developed disease. The authors also demonstrated that the gut microbiota composition was changed by MyD88 deficiency and colonization of GF MyD88-deficient NOD mice with the bacterial community termed “altered Schedler’s flora” (ASF), normally present in healthy mouse gut, attenuated T1D. Thus, it can be suggested that in the absence of TLRs signaling, some bacterial groups predominate and induce tolerogenic responses. However, the receptors and signaling pathways involved in microbiota-dependent protection against T1D development remain unclear.

Some protective signals against T1D development triggered by the gut microbiota have been revealed through studies with NOD mice lacking MyD88 crossed with mice deficient for other components of the innate immune response. As ASF bacteria colonization has reduced T1D in GF MyD88-deficient NOD mice, it is possible that several signaling pathways activated by TLR agonists could contribute to protection when MyD88 signaling is absent. In fact, stimulation of TLR3 and TLR4 induces the activation of a MyD88-independent, TRIF-dependent signaling pathway (55), which has been pointed out as a negative regulator of immunity. In this context, Burrows and colleagues (92) have demonstrated that TRIF deficiency did not affect T1D development in Myd88 sufficient NOD mice, while double-deficient mice were more susceptible to T1D. Thus, it could be suggested that TLR3- and TLR4-mediated sensing of the gut microbiota participates in the protection against T1D development in MyD88-deficient NOD mice through tolerating mechanisms involving TRIF signaling.

Protective effects from TLR–microbiota interaction have also been demonstrated in MS studies. The MS is an immune-mediated chronic disease in which demyelination and axonal damage are caused by infiltration of myelin-specific autoreactive Th1 and Th17 cells into the central nervous system (CNS) in either relapsing/remitting or progressive condition (93, 94). CNS-derived peptides, such as the melanocortin antagonist agouti-related peptide (AgRP) and neuropeptide Y (NPY), are capable of modulating food intake and physiological processes that control nutrient absorption, which may influence the gut microbiota composition. In turn, gut microbiota modulates brain functions by releasing SCFAs and antigens, such as lipopolysaccharide (LPS), polysaccharide A (PSA), and LTA (95).

Miyake and colleagues (96) have investigated whether the gut microbiota was altered in MS by comparing the gut microbiota of 20 patients with relapsing–remitting (RR) MS with that of 40 healthy subjects. They found differences in the relative abundance of both archaea and butyrate-producing bacteria when comparing MS patients and healthy individuals and a significant reduction of clostridial species in MS patients. Interestingly, none of the clostridial species that were significantly reduced in the gut microbiota of MS patients overlapped with other spore-forming clostridial species capable of inducing colonic regulatory T cells, which have been associated with protection from autoimmunity and allergies (97). However, it has been shown that reconstitution of the gut microbiota from antibiotic-treated mice with *Bacteroides fragilis* protected against experimental autoimmune encephalomyelitis (EAE) by a mechanism dependent on PSA-induced IL-10-producing Treg cells, which may rely on TLR2 signaling (98). In fact, *B. fragilis*-derived PSA converts effector CD4^+^ T cells into IL-10-producing T cells *in vitro* by a TLR2-dependent mechanism (99). On the other hand, TLR4-mediated sensing of LPS has been shown to stimulate *in vitro* differentiation of Th17 cells and subcutaneous LPS injection increased the frequency of IL-17-producing cells in inguinal lymph nodes, worsening EAE (100).

Numerous studies have focused on the role of B cells during MS pathogenesis (101, 102). B cell-deficient C57Bl/6 mice suffer an exacerbate EAE form and transfer of IL-10-producing B cells into IL-10-deficient mice protected them from disease (101). In fact, it has been recently shown that microbiota-driven IL-1β and IL-6 production promotes regulatory B cells differentiation (103). Lampropoulou et al. (102) showed that TLR-activated B cells produce IL-10 in a MyD88-dependent manner by stimulating TLR4 and TLR9 with LPS and CpG oligonucleotides, respectively. These B cells limit the capacity of DC to produce IL-12 and restrain Th1 differentiation. Therefore, new studies are fundamental to elucidate the role of different TLRs in the context of EAE modulation, which seems to depend on microbiota-driven inflammatory and regulatory immune cells.

## Inflammasomes in the Gut

Inflammasomes are multimeric protein complexes typically composed of a sensor protein, the adaptor protein ASC, and the proinflammatory caspase-1, which can be triggered by a variety of stimuli associated with infection and cellular stress (104). Inflammasome activation results in recruitment of ASC, proteolytic cleavage, and activation of caspase-1, which leads to process and release of the proinflammatory cytokines IL-1β and IL-18 (105). The majority of inflammasomes contain a NLR sensor molecule, namely NLRP1 (NOD-, LRR-, and pyrin domain-containing 1), NLRP3, NLRP6, NLRP7, NLRP12, or NLRC4 (NOD-, LRR-, and CARD-containing 4). However, other two inflammasomes have been described containing the pyrin and HIN domain-containing protein (PYHIN) family members absent in melanoma 2 (AIM2) and IFN-γ-inducible protein 16 (IFI16) as sensor molecules (106). A non-canonical activation pathway of inflammatory caspases 4/5 in humans and caspase-11 in mice has also been described and depends on intracellular sensing of lipopolysaccharide (LPS) released upon Gram-negative bacteria escape from vacuoles (107).

Inflammasome signaling has been extensively studied in macrophages in different contexts (104, 108), but little is known about inflammasome expression and function in cells located in the gut. Intestinal CD11b^+^F4/80^+^ mononuclear phagocytes that normally reside in the lamina propria were shown to be anergic to ligands for TLRs or commensals but to produce IL-1β upon NLRC4 activation after infection with pathogenic *Salmonella* or *Pseudomonas* (109). More recently, *Enterobacteriaceae* and the pathobiont *Proteus mirabilis* were shown to induce robust IL-1β production through NLRP3 activation in newly recruited intestinal Ly6C^high^ monocytes upon epithelial injury (110). IECs, the first cellular barrier toward the gut lumen, were also shown to express a variety of inflammasome components such as NAIPs 1, 2, 5, and 6 in mice (hNAIP in humans), NLRC4, NLRP1, NLRP6, AIM2, caspase-1, caspase-11 (-4, in humans), ASC, and IL-18. These inflammasome components contribute to intestinal homeostasis by regulating commensals’ ecology, by restricting pathogens, and by restoring epithelial barrier integrity (105). However, further studies are still necessary to shed light on how inflammasomes are regulated in both hematopoietic and non-hematopoietic intestinal cells in face of commensal microorganisms, diet-derived antigens, and pathogens to maintain homeostasis and systemic immunity.

## Inflammasome-Mediated Sensing of the Gut Microbiota and Inflammation

The NLRP6 inflammasome has been shown to regulate intestinal microbiota ecology as metagenomic analysis has revealed pronounced dysbiosis in NLRP6-deficient mice (111, 112). Changes in the biogeographical distribution of microbiota are also observed in NLRP6 deficiency, leading to accumulation of commensal microorganisms in the colonic crypts. Reduced IL-18 levels and increased relative abundance of bacterial phyla Bacteroidetes (*Prevotellaceae*) and TM7 have been observed in NLRP6-deficient mice, which present spontaneous intestinal hyperplasia, chemokine (C–C motif) ligand 5 (CCL5)-mediated inflammatory cell recruitment, and exacerbation of DSS-induced colitis. Cross-fostering and cohousing experiments were sufficient to transfer dysbiotic microbiota to neonatal and adult wild-type (WT) mice, leading to increased susceptibility to DSS-induced colitis. Although deficiency in other inflammasome components, including ASC, caspase-1, and IL-18, also led to dysbiosis and exacerbated colitis, deficiency in NLRC4, NLRP10, NLRP12, and AIM2 had no impact on the susceptibility of WT mice to colitis upon cohousing (111). These mice were shown to have a distinct configuration of their microbiota when compared to NLRP6-deficient mice, which might explain the different results and suggests that although inflammasomes share many of their effector molecules, different impacts on microbiota ecology can be observed.

It has been recently shown that mice deficient in NLRP6 and the inflammasome components ASC and caspase-1 lack a thick continuous overlaying inner mucus layer in the gut due to abrogated mucus secretion by IECs and are unable to clear enteric pathogens from mucosal surface (113). As mucus has an important role in regulating host–microbial interactions, it is likely that dysbiosis and increased intestinal inflammation observed in NLRP6 mice are consequences of this reduced mucus secretion by epithelial cells. Deficiency in NLRP6 and IL-18 has also been linked to colitis-related colorectal cancer (CRC) development. Enhanced tumorigenesis in these mice was dependent on microbiota-induced CCL5-driven inflammation and local IL-6 production and could be transferred to WT mice upon cohousing (114).

Corticotropin-releasing hormone (CRH)-mediated reduction of intestinal NLRP6 expression in mice exposed to water-avoidance stress (WAS) has been shown to result in altered gut microbiota and acute small intestinal inflammation. These mice presented intestinal erythema, leukocyte infiltration, increased intestinal permeability, and increased mucosal expression of IL-17 and IL-6. Other inflammasome components that had their expression partially inhibited by WAS were ASC, caspase-1, IL-1β, and IL-18, while no significant impact was observed on NLRP3 expression. As observed for colitis and colitis-related CRC, non-stressed mice developed enteritis upon cohousing with WAS-exposed mice and probiotic therapy prior to WAS reduced intestinal inflammation and prevented WAS-mediated dysbiosis (115).

NLRP3-deficient mice have also been shown to be more susceptible to DSS-induced colitis, to have a dysbiotic microbiota, and to present altered colonic β-defensin expression and decreased antimicrobial capacity. Their neutrophils exhibited impaired chemotaxis and enhanced spontaneous apoptosis (116). Modulation of NLRP3 in the gut epithelium by high-fiber feeding has been suggested to contribute to intestinal homeostasis and protection from colitis through maintenance of a healthy microbiota and due to SCFA-mediated sensing by G-protein-coupled receptors GPR43 and GPR109A and IL-18 release (117). However, upon epithelial injury, members of the gut microbiota, such as *Enterobacteriaceae*, and in particular the pathobiont *P. mirabilis* induce NLRP3-mediated IL-1β release by Ly6C^high^ monocytes, leading to intestinal inflammation (110).

The influence of inflammasome-mediated sensing of the microbiota goes beyond inflammatory processes in the gut. NLRP3, NLRP6, and inflammasome components ASC, caspase-1, and IL-18-deficiency-associated dysbiosis leads to exacerbated hepatic steatosis and inflammation through influx of TLR4 and TLR9 agonists into the portal circulation, which results in enhanced hepatic TNF-α expression and non-alcoholic steatohepatitis (NASH)/non-alcoholic fatty liver disease (NAFLD) progression. Cohousing experiments were sufficient to exacerbate NASH in WT mice, suggesting that intestinal dysbiosis may govern initial steps of systemic autoinflammatory disorders (118).

It has been shown that Pstpip2^cmo^ mice, which are prone to develop osteomyelitis, present dysbiotic intestinal microbiota characterized by *Prevotella* outgrowth. Interestingly, these mice were protected from inflammatory bone disease and bone erosion when fed with high-fat diet (HFD), which had a marked impact on intestinal *Prevotella* reduction and significantly reduced pro-IL-1β expression in neutrophils. Antibiotic treatment was also efficient in reducing pro-IL-1β expression in Pstpip2^cmo^ mice, but cohousing experiments and fecal microbiota transplantation from Pstpip2^cmo^ mice failed to cause disease in WT mice. However, fecal microbiota transplantation from low-fat diet (LFD)-fed diseased Pstpip2^cmo^ mice to young LFD-fed Pstpip2^cmo^ mice by oral gavage promoted the expansion of *Prevotella*, and significantly accelerated the development of osteomyelitis, whereas transplantation of fecal microbiota from HFD-fed Pstpip2^cmo^ mice to young LFD-fed Pstpip2^cmo^ mice limited *Prevotella* outgrowth and significantly protected mice from developing osteomyelitis. Caspase-1/8-mediated processing of microbiota-induced pro-IL-1β was necessary to promote autoinflammatory disease in these susceptible mice (119).

Extraintestinal inflammatory processes have also been shown to be partially dependent on inflammasome-mediated sensing of the microbiota metabolite acetate. GF, antibiotic-treated, and GPR43-dificient mice are protected from joint inflammation upon injection of monosodium urate monohydrate (MSU) crystals. It was demonstrated that microbiota reconstitution or acetate administration to GF mice restored MSU crystals-induced inflammation and that GPR43 is at least partially necessary to adequate inflammasome assembly and IL-1β production in response to acetate (120). Therefore, one may suggest that inflammasome-mediated sensing of both intestinal microbiota and their products contributes to control local and systemic inflammatory disorders, which should be considered when designing prophylactic and therapeutic applications.

## Inflammasome and TLR Interactions

Many studies have focused on the interactions between inflammasomes and TLRs. In fact, it has been shown that the enteric pathogens *E. coli*- and *Citrobacter rodentium*-induced TLR4/TRIF-dependent synthesis of caspase-11 and activation of NLRP3 inflammasome in macrophages (121) and that *Pseudomonas aeruginosa* infection of macrophages induced TLR-4/TRIF-dependent autophagy, which was attenuated by NLRC4/caspase-1-mediated cleavage of TRIF. Prevention of *in vivo* caspase-1-mediated cleavage of TRIF resulted in enhanced autophagy, reduced IL-1β production, and increased bacterial clearance (122). *L. monocytogenes* infection of macrophages was shown to induce TLR/IRAK1/IRAK4-dependent activation of NLRP3 (123, 124), which suggests that inflammasome–TLR interactions may have an important role in the bacterial recognition.

It has been also demonstrated that Clathrin-mediated endocytosis followed by TLR8- and TLR7-mediated recognition of HIV and HCV in monocytes and macrophages resulted in NLRP3 activation, which was independent of type I IFN production (125). Moreover, dendritic cells were shown to express higher levels of NLRP3 in the steady-state condition compared to macrophages and to secrete substantial amounts of mature IL-1β upon stimulation with TLR ligands independently of P2X7 signaling (126). On the other hand, chronic TLR stimulation by LPS has been shown to dampen NLRP3 activation through IL-10 induction (127), and TLR2/TLR4 engagement resulted in upregulation of plasminogen activator inhibitor type 2 (PAI-2), a serine protease inhibitor, which culminated in the stabilization of the autophagic protein Beclin 1 to promote autophagy, reduction of mitochondrial reactive oxygen species, NLRP3 protein level, and pro-IL-1β processing (128).

Although increasing evidence suggests that both TLRs and inflammasomes play an important role in promoting host–microbiota communication, studies regarding the interactions of these molecules in the context of the gut microbiota are still lacking and will further improve our knowledge on how commensal microorganisms and the immune system cooperate in the modulation of inflammatory and autoimmune diseases.

## Concluding Remarks

The dynamic interactions that have been established between mammalian hosts and commensal microorganisms over millennia of coevolution resulted in the development of a well-structured immune system, which controls pathogenic infections while tolerates a highly diverse microbiota. Innate sensing of the gut microbiota through TLRs and inflammasomes contributes to intestinal homeostasis by stimulating the development/function of both regulatory and inflammatory cells and by promoting proliferation of IECs, epithelial integrity, mucus secretion, and containment of opportunistic infections. It has become clear that disturbances in the fine-tuning that governs host–microbiota interactions may lead to both local and systemic inflammatory and autoimmune diseases. In this review, we bring many examples of the dualistic role played by the gut microbiota in both promoting and regulating inflammation and autoimmunity. We believe that a better understanding of mechanisms involved in these complex interactions between host and commensals will further improve common prophylactic and therapeutic applications.

## Author Contributions

AI, CM, and RA wrote the manuscript. NC and RA reviewed the manuscript.

## Conflict of Interest Statement

The authors declare that the research was conducted in the absence of any commercial or financial relationships that could be construed as a potential conflict of interest.
